# Crystal structure of (2*E*)-1-(5-bromo­thio­phen-2-yl)-3-(2-chloro­phen­yl)prop-2-en-1-one

**DOI:** 10.1107/S2056989015021155

**Published:** 2015-11-14

**Authors:** B. R. Anitha, M. Vinduvahini, A. J. Ravi, H. C. Devarajegowda

**Affiliations:** a Department of Physics, Yuvaraja’s College (Constituent College), University of Mysore, Mysore 570 005, Karnataka, India; b Department of Physics, Sri D Devaraja Urs Govt. First Grade College, Hunsur 571 105, Mysore District, Karnataka, India

**Keywords:** crystal structure, chalcone, π–π inter­actions

## Abstract

In the title compound, C_13_H_8_BrClOS, the thienyl ring is not coplanar with the benzene ring, their planes forming a dihedral angle of 13.2 (4)°. In the crystal, mol­ecules stack along the *a* axis, with the inter­planar separation between thienyl rings and between benzene rings being 3.925 (6) Å. The sample is an inversion twin.

## Related literature   

For general background to chalcones, see: Lin *et al.* (2001 ▸); Horng *et al.* (2003 ▸); López *et al.* (2001 ▸); Liu *et al.* (2003 ▸). For related crystal structures, see: Liang *et al.* (2011 ▸); Alex *et al.* (1993 ▸); Li & Su (1993 ▸).
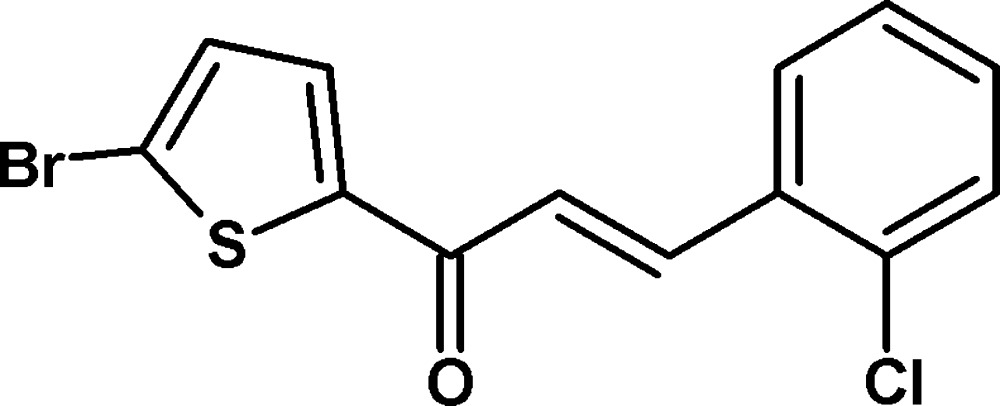


## Experimental   

### Crystal data  

C_13_H_8_BrClOS
*M* *_r_* = 327.61
Orthorhombic, 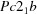

*a* = 3.9247 (19) Å
*b* = 11.549 (6) Å
*c* = 28.111 (14) Å
*V* = 1274.1 (11) Å^3^
*Z* = 4
Mo *K*α radiation
μ = 3.58 mm^−1^
*T* = 293 K
0.24 × 0.20 × 0.12 mm

### Data collection  

Bruker SMART CCD area-detector diffractometer
Absorption correction: ψ scan (*SADABS*; Sheldrick, 2007 ▸) *T*_min_ = 0.770, *T*_max_ = 1.000
12995 measured reflections
3007 independent reflections
2096 reflections with *I* > 2σ(*I*)
*R*_int_ = 0.065

### Refinement  

*R*[*F* ^2^ > 2σ(*F* ^2^)] = 0.062
*wR*(*F* ^2^) = 0.137
*S* = 1.10
3007 reflections
155 parameters
1 restraint
H-atom parameters constrained
Δρ_max_ = 0.79 e Å^−3^
Δρ_min_ = −0.33 e Å^−3^
Absolute structure: refined as an inversion twin
Absolute structure parameter: 0.15 (3)

### 

Data collection: *SMART* (Bruker, 2001 ▸); cell refinement: *SAINT* (Bruker, 2001 ▸); data reduction: *SAINT*; program(s) used to solve structure: *SHELXS97* (Sheldrick, 2008 ▸); program(s) used to refine structure: *SHELXL2014* (Sheldrick, 2015 ▸); molecular graphics: *ORTEP-3 for Windows* (Farrugia, 2012 ▸); software used to prepare material for publication: *SHELXL2014*.

## Supplementary Material

Crystal structure: contains datablock(s) I, global. DOI: 10.1107/S2056989015021155/tk5406sup1.cif

Structure factors: contains datablock(s) I. DOI: 10.1107/S2056989015021155/tk5406Isup2.hkl

Supporting information file. DOI: 10.1107/S2056989015021155/tk5406Isup3.cml

**Fig. 1.**
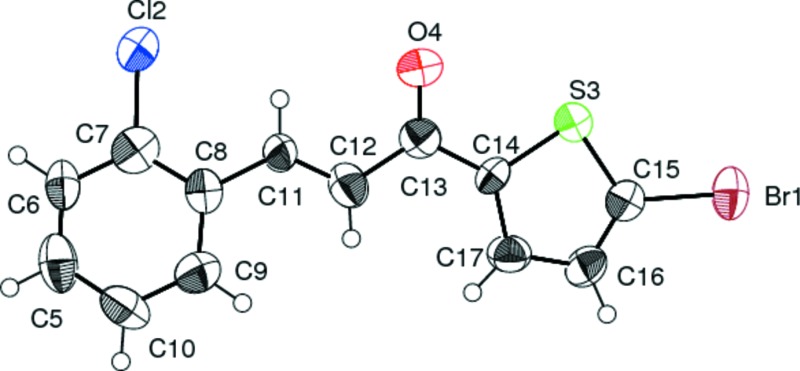
The molecular structure of the title compound, with displacement ellipsoids drawn at the 50% probability level.

. DOI: 10.1107/S2056989015021155/tk5406fig1.tif
The mol­ecular structure of the title compound, with displacement ellipsoids drawn at the 50% probability level.

CCDC reference: 1435865

Additional supporting information:  crystallographic information; 3D view; checkCIF report

## supplementary crystallographic information

### S1. Comment

Chalcones are alpha,beta unsaturated ketones, widely distributed in nature and are extensively studied for their biological activity (Lin *et al.*, 2001; Horng *et al.*, 2003; López *et al.*, 2001; Liu *et al.*, 2003). We report here the crystal structure of a bromo derivative of hetero aryl chalcone which has shown aldose reductase inhibition in the virtual screening study conducted by us.

The asymmetric unit of (2E)-1-(5-bromo-2-thienyl)-3-(2-chlorophenyl) prop-2-en-1-one, C_13_H_8_Br Cl O S, contains just one molecule (Fig. 1). The five-membered thiophene ring (S2,C5–C8) is not coplanar with the benzene ring (C12–C17); the dihedral angle between the two planes is 13.2 (4)°. Bond lengths and angles are in good agreement with those observed in related crystal structures (Liang *et al.*, 2011; Alex *et al.*, 1993; Li *et al.*, 1993).

### S2. Experimental

A mixture of 2-acetyl-5-bromothiophene (0.01 mol) and 2-chlorobenzaldehyde (0.01 mol) were stirred in ethanol (30 ml) and then an aqueous solution of potassium hydroxide (40%,15 ml) was added. The mixture was kept over night at room temperature, poured into crushed ice and acidified with dilute hydrochloric acid. The precipitated chalcone was filtered and crystallized from ethanol.

### S3. Refinement

All H atoms were positioned at calculated positions with C—H = 0.93 Å, and with *U*_iso_(H) = 1.2*U*_eq_(C). The sample was refined as an inversion twin.

### Crystal data

**Table:**

C_13_H_8_BrClOS	*D*_x_ = 1.708 Mg m^−^^3^
*M**_r_* = 327.61	Melting point: 342 K
Orthorhombic, *P**c*2_1_*b*	Mo *K*α radiation, λ = 0.71073 Å
*a* = 3.9247 (19) Å	Cell parameters from 3007 reflections
*b* = 11.549 (6) Å	θ = 2.3–28.0°
*c* = 28.111 (14) Å	µ = 3.58 mm^−^^1^
*V* = 1274.1 (11) Å^3^	*T* = 293 K
*Z* = 4	Prism, colourless
*F*(000) = 648	0.24 × 0.20 × 0.12 mm

### Data collection

**Table:**

Bruker SMART CCD area-detector diffractometer	3007 independent reflections
Radiation source: fine-focus sealed tube	2096 reflections with *I* > 2σ(*I*)
Graphite monochromator	*R*_int_ = 0.065
ω and φ scans	θ_max_ = 28.0°, θ_min_ = 2.3°
Absorption correction: ψ scan (*SADABS*; Sheldrick, 2007)	*h* = −5→5
*T*_min_ = 0.770, *T*_max_ = 1.000	*k* = −15→14
12995 measured reflections	*l* = −37→36

### Refinement

**Table:**

Refinement on *F*^2^	Hydrogen site location: inferred from neighbouring sites
Least-squares matrix: full	H-atom parameters constrained
*R*[*F*^2^ > 2σ(*F*^2^)] = 0.062	*w* = 1/[σ^2^(*F*_o_^2^) + (0.0565*P*)^2^ + 0.585*P*] where *P* = (*F*_o_^2^ + 2*F*_c_^2^)/3
*wR*(*F*^2^) = 0.137	(Δ/σ)_max_ < 0.001
*S* = 1.10	Δρ_max_ = 0.79 e Å^−^^3^
3007 reflections	Δρ_min_ = −0.33 e Å^−^^3^
155 parameters	Absolute structure: refined as an inversion twin
1 restraint	Absolute structure parameter: 0.15 (3)

### Special details

**Table:**

Geometry. All e.s.d.'s (except the e.s.d. in the dihedral angle between two l.s. planes) are estimated using the full covariance matrix. The cell e.s.d.'s are taken into account individually in the estimation of e.s.d.'s in distances, angles and torsion angles; correlations between e.s.d.'s in cell parameters are only used when they are defined by crystal symmetry. An approximate (isotropic) treatment of cell e.s.d.'s is used for estimating e.s.d.'s involving l.s. planes.
Refinement. Refined as a 2-component inversion twin.

### Fractional atomic coordinates and isotropic or equivalent isotropic displacement parameters (Å^2^)

**Table:**

	*x*	*y*	*z*	*U*_iso_*/*U*_eq_	
Br1	0.8042 (2)	0.11487 (13)	0.17078 (3)	0.0615 (4)	
S2	0.5184 (8)	0.2199 (2)	0.07773 (9)	0.0516 (7)	
Cl2	−0.0696 (11)	0.3138 (3)	−0.17931 (10)	0.0789 (10)	
O4	0.200 (2)	0.2992 (7)	−0.0100 (3)	0.072 (2)	
C5	0.617 (2)	0.1001 (11)	0.1095 (3)	0.045 (2)	
C6	0.541 (3)	0.0007 (10)	0.0881 (4)	0.056 (3)	
H6	0.5781	−0.0720	0.1014	0.068*	
C7	0.399 (3)	0.0198 (10)	0.0433 (4)	0.055 (3)	
H7	0.3336	−0.0393	0.0228	0.066*	
C8	0.367 (2)	0.1372 (9)	0.0325 (3)	0.040 (2)	
C9	0.224 (2)	0.1930 (9)	−0.0097 (4)	0.047 (2)	
C10	0.115 (2)	0.1212 (13)	−0.0502 (3)	0.056 (2)	
H10	0.1262	0.0410	−0.0477	0.067*	
C11	0.004 (3)	0.1676 (9)	−0.0897 (3)	0.050 (3)	
H11	−0.0011	0.2481	−0.0904	0.060*	
C12	−0.1123 (19)	0.1090 (13)	−0.1328 (3)	0.047 (2)	
C13	−0.161 (2)	0.1657 (10)	−0.1751 (4)	0.056 (3)	
C14	−0.284 (2)	0.1127 (16)	−0.2156 (3)	0.059 (2)	
H14	−0.3209	0.1551	−0.2433	0.071*	
C15	−0.351 (3)	−0.0028 (14)	−0.2143 (4)	0.071 (3)	
H15	−0.4279	−0.0402	−0.2415	0.085*	
C16	−0.305 (3)	−0.0644 (12)	−0.1730 (4)	0.070 (3)	
H16	−0.3591	−0.1428	−0.1723	0.084*	
C17	−0.179 (3)	−0.0115 (10)	−0.1326 (4)	0.065 (3)	
H17	−0.1375	−0.0550	−0.1053	0.077*	

### Atomic displacement parameters (Å^2^)

**Table:**

	*U*^11^	*U*^22^	*U*^33^	*U*^12^	*U*^13^	*U*^23^
Br1	0.0533 (5)	0.0886 (8)	0.0426 (5)	0.0105 (8)	−0.0063 (4)	−0.0005 (9)
S2	0.0619 (19)	0.0474 (14)	0.0454 (14)	0.0001 (14)	−0.0050 (14)	−0.0022 (12)
Cl2	0.114 (3)	0.0636 (19)	0.0588 (18)	−0.0100 (19)	−0.0207 (17)	0.0143 (15)
O4	0.105 (6)	0.045 (5)	0.067 (5)	−0.003 (4)	−0.014 (4)	0.005 (4)
C5	0.043 (4)	0.049 (6)	0.043 (4)	0.002 (6)	0.003 (3)	0.003 (5)
C6	0.060 (7)	0.050 (7)	0.060 (7)	0.013 (6)	−0.009 (6)	0.012 (5)
C7	0.063 (7)	0.040 (6)	0.063 (7)	0.004 (5)	−0.004 (5)	−0.002 (5)
C8	0.038 (5)	0.046 (7)	0.036 (4)	0.002 (4)	0.001 (3)	0.008 (4)
C9	0.039 (6)	0.049 (7)	0.054 (6)	−0.006 (4)	−0.002 (4)	0.004 (5)
C10	0.056 (5)	0.059 (6)	0.053 (5)	0.006 (7)	−0.005 (4)	−0.010 (7)
C11	0.059 (7)	0.048 (5)	0.043 (5)	−0.016 (4)	−0.012 (5)	0.005 (4)
C12	0.037 (4)	0.063 (6)	0.041 (4)	0.001 (7)	0.004 (3)	−0.002 (6)
C13	0.042 (5)	0.065 (7)	0.062 (7)	0.005 (5)	0.012 (5)	0.005 (5)
C14	0.063 (6)	0.075 (7)	0.039 (4)	0.006 (8)	−0.009 (4)	−0.004 (9)
C15	0.069 (8)	0.110 (11)	0.034 (6)	0.007 (7)	0.002 (5)	−0.012 (6)
C16	0.074 (8)	0.059 (8)	0.077 (9)	−0.015 (6)	0.002 (7)	−0.023 (7)
C17	0.073 (7)	0.055 (7)	0.066 (7)	−0.007 (6)	−0.004 (6)	0.010 (6)

### Geometric parameters (Å, º)

**Table:**

Br1—C5	1.880 (9)	C10—H10	0.9300
S2—C5	1.691 (11)	C11—C12	1.462 (13)
S2—C8	1.697 (10)	C11—H11	0.9300
Cl2—C13	1.750 (12)	C12—C13	1.370 (14)
O4—C9	1.230 (12)	C12—C17	1.417 (19)
C5—C6	1.329 (16)	C13—C14	1.382 (15)
C6—C7	1.397 (14)	C14—C15	1.36 (3)
C6—H6	0.9300	C14—H14	0.9300
C7—C8	1.395 (16)	C15—C16	1.374 (17)
C7—H7	0.9300	C15—H15	0.9300
C8—C9	1.462 (13)	C16—C17	1.381 (16)
C9—C10	1.473 (15)	C16—H16	0.9300
C10—C11	1.306 (13)	C17—H17	0.9300
			
C5—S2—C8	90.9 (5)	C10—C11—H11	115.9
C6—C5—S2	114.6 (7)	C12—C11—H11	115.9
C6—C5—Br1	125.4 (9)	C13—C12—C17	116.6 (10)
S2—C5—Br1	120.0 (7)	C13—C12—C11	122.8 (12)
C5—C6—C7	111.2 (10)	C17—C12—C11	120.6 (10)
C5—C6—H6	124.4	C12—C13—C14	123.5 (13)
C7—C6—H6	124.4	C12—C13—Cl2	119.8 (10)
C8—C7—C6	112.7 (10)	C14—C13—Cl2	116.7 (10)
C8—C7—H7	123.7	C15—C14—C13	118.7 (11)
C6—C7—H7	123.7	C15—C14—H14	120.7
C7—C8—C9	129.7 (9)	C13—C14—H14	120.7
C7—C8—S2	110.7 (7)	C14—C15—C16	120.4 (10)
C9—C8—S2	119.6 (8)	C14—C15—H15	119.8
O4—C9—C8	118.4 (10)	C16—C15—H15	119.8
O4—C9—C10	122.2 (10)	C15—C16—C17	120.8 (12)
C8—C9—C10	119.5 (10)	C15—C16—H16	119.6
C11—C10—C9	121.5 (13)	C17—C16—H16	119.6
C11—C10—H10	119.2	C16—C17—C12	119.8 (11)
C9—C10—H10	119.2	C16—C17—H17	120.1
C10—C11—C12	128.2 (12)	C12—C17—H17	120.1
			
C8—S2—C5—C6	−0.3 (8)	C9—C10—C11—C12	−179.9 (9)
C8—S2—C5—Br1	−178.3 (5)	C10—C11—C12—C13	−166.9 (10)
S2—C5—C6—C7	0.8 (12)	C10—C11—C12—C17	12.5 (16)
Br1—C5—C6—C7	178.7 (7)	C17—C12—C13—C14	3.3 (14)
C5—C6—C7—C8	−1.1 (14)	C11—C12—C13—C14	−177.3 (9)
C6—C7—C8—C9	−178.2 (10)	C17—C12—C13—Cl2	−177.6 (8)
C6—C7—C8—S2	0.9 (12)	C11—C12—C13—Cl2	1.9 (13)
C5—S2—C8—C7	−0.4 (8)	C12—C13—C14—C15	−2.4 (15)
C5—S2—C8—C9	178.8 (8)	Cl2—C13—C14—C15	178.5 (8)
C7—C8—C9—O4	175.0 (10)	C13—C14—C15—C16	1.8 (17)
S2—C8—C9—O4	−4.0 (13)	C14—C15—C16—C17	−2.3 (19)
C7—C8—C9—C10	−5.4 (16)	C15—C16—C17—C12	3.3 (18)
S2—C8—C9—C10	175.6 (7)	C13—C12—C17—C16	−3.7 (15)
O4—C9—C10—C11	3.5 (16)	C11—C12—C17—C16	176.8 (10)
C8—C9—C10—C11	−176.1 (9)		
