# Supplementary figures and images for: Crystal structure of (2E)-1-(5-bromo­thio­phen-2-yl)-3-(2-chloro­phen­yl)prop-2-en-1-one
Source: Acta Crystallogr E Crystallogr Commun. 2015 Nov 14;71(Pt 12):o930. doi: 10.1107/S2056989015021155 (PMC4719886; doi:10.1107/S2056989015021155)

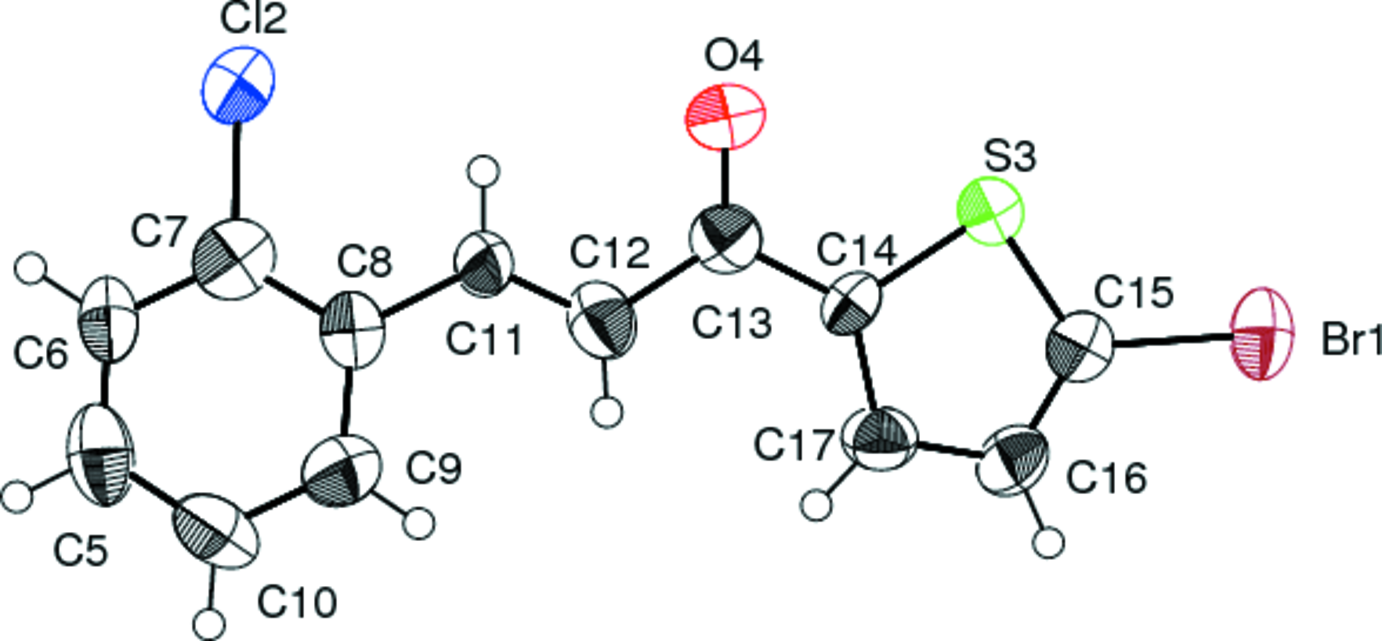

Supplement: Supplementary file 4 [file e-71-0o930-fig1.tif]
